# Using Artificial Intelligence for Drug Discovery: A Bibliometric Study and Future Research Agenda

**DOI:** 10.3390/ph15121492

**Published:** 2022-11-30

**Authors:** Erik Karger, Marko Kureljusic

**Affiliations:** 1 Information Systems and Strategic IT Management, University of Duisburg-Essen, 45141 Essen, Germany; 2 International Accounting, University of Duisburg-Essen, 45141 Essen, Germany

**Keywords:** drug discovery, drug development, artificial intelligence, machine learning, deep learning, bibliometric study

## Abstract

Drug discovery is usually a rule-based process that is carefully carried out by pharmacists. However, a new trend is emerging in research and practice where artificial intelligence is being used for drug discovery to increase efficiency or to develop new drugs for previously untreatable diseases. Nevertheless, so far, no study takes a holistic view of AI-based drug discovery research. Given the importance and potential of AI for drug discovery, this lack of research is surprising. This study aimed to close this research gap by conducting a bibliometric analysis to identify all relevant studies and to analyze interrelationships among algorithms, institutions, countries, and funding sponsors. For this purpose, a sample of 3884 articles was examined bibliometrically, including studies from 1991 to 2022. We utilized various qualitative and quantitative methods, such as performance analysis, science mapping, and thematic analysis. Based on these findings, we furthermore developed a research agenda that aims to serve as a foundation for future researchers.

## 1. Introduction

### 1.1. Motivation

Diseases and sickness can be defined as conditions that negatively affect an organism and its functions. Diseases can affect most living organisms, including humans. Understanding the nature to develop new drugs and medicine to fight diseases is therefore a goal that is as old as human civilization itself [1]. Life expectancy, one of the key metrics to assess the health of a population, increased significantly over the last decades [2]. There is a debate about what the reasons for the increase in life expectancy are, as hygiene and nutrition also improved. However, there is little doubt that advances in modern medicine and the development of new drugs played a key role in fighting and controlling infectious diseases [3].

The process of drug discovery and its underlying paradigms were subject to several changes and developments over the last centuries. This process went from a trial-and-error method of natural products to the development of synthetic, biotechnological, or biopharmaceutical drugs [1]. The instruments and tools that are used for the process of drug discovery did change significantly. Especially in the last decades, digital technologies were increasingly applied in the process of drug discovery. Examples of digital technologies in pharmacy are the usage of laboratory robotics [4] or automation in medicinal chemistry [5]. One of the most discussed technologies of the digital age that is also increasingly used for medical purposes is artificial intelligence (AI). “AI” is a trending term that has, to the best of our knowledge, no precise and generally accepted definition [6]. In general terms, AI refers to the approach of simulating intelligence with computers [7]. It aims to understand and replicate cognitive processes and relies on principles and input from many different disciplines, including mathematics, biology, and engineering [8].

Recent advances in technology and the availability of new hardware that allow fast parallel processing have made AI a technology that is applicable to many real-world applications [9]. Additionally, data that are necessary to train AI systems have become more easily available. PubChem is probably one of the most known public repositories and contains information on chemical substances, as well as experimental data identifying the biological activities of these molecules [10,11]. Driven by these developments, the application of AI technologies within drug discovery grew significantly in the last decade. Established AI and machine learning methods such as support vector machines (SVM) [12,13] and artificial neural networks [14,15] were increasingly used to discover and understand drugs and their properties. Especially in the last few years, deep learning evolved a lot and proved to have an advantage over other machine learning technologies in many areas [16]. Deep learning has also been successfully applied in drug discovery [17], for example, to predict pharmaceutical properties [18] or for the discovery of antibiotics [19]. AI’s high potential is also reflected in the AI in pharma market, which experienced strong growth in the last years. Companies such as Microsoft, IBM Watson, Google, and Novartis are among the major players participating in it. Until 2026, AI in pharma is forecasted to have a market value of USD 3626 million US, with a compound annual growth rate of 30.9% [20].

Especially within the last five years, research on AI for drug discovery has grown rapidly. It is nowadays a research field that consists of several contributions from scholars of many different disciplines. For scholars and practitioners that are interested in that field, it is hard to oversee all the different contributions and key issues that are addressed. With this article, we aim to extend the literature and research on AI for drug discovery by identifying the core topics, the most influential institutions and funding sponsors, and the current development of this research field. We believe this is necessary to consolidate existing contributions to provide both research and practice with a summarized overview of AI for drug discovery. The first research question we aim to address is as follows:

*RQ1:* What is the present status of research on using AI for drug discovery, and what topics have been investigated in previous research?

Additionally, an article that analyzes the prior literature can “provide directions for future research with reference to new and novel ideas, theories, measures, methods and novel research questions” [21] (p. 1). We follow [21] and believe that review articles, including bibliometric studies, can and should identify future research questions to advance a certain research field. Our second goal is therefore to serve as a foundation for interested scholars by identifying opportunities for further research. Hence, we aim to address the following second research question:

*RQ2:* What are promising future research avenues that can help to advance the field of AI for drug discovery?

To answer the first research question, we followed a bibliometric approach. While systematic literature reviews are used to qualitatively analyze smaller datasets of literature, bibliometric studies aim at quantitatively analyzing large datasets by using statistical or visualization tools [22]. Hereby, a bibliometric analysis can help to achieve a comprehensive understanding of a research field and its boundaries and can furthermore help to identify future research directions [23,24,25,26]. Although the bibliometric method is not new and was discussed already in the 1950s and 1960s [27,28], it has gained popularity in recent years. Due to their benefits and value, bibliometric studies have been applied in different disciplines, including pharmacy [29,30], oncology [31], tourism management [32], human resources management [33], and business administration [34]. Given a large amount of research available on that topic, we found a bibliometric approach also suitable for the field of AI for drug discovery.

While we used the bibliometric analysis to understand the structure and research topics, we followed [23] and additionally applied content analysis. Similar approaches have already been conducted by different researchers [23,32,35,36] to obtain more detailed insights. In our study, we used additional content analysis to identify promising future research topics to answer our second research question.

### 1.2. Overview of Artificial Intelligence

AI is one of the newest fields that is investigated in science and engineering [37]. According to [38], the beginning of AI can be dated to 1943. Going back to this year, [39] proposed the first idea of an artificial neuron. The term “AI” itself was coined a few years later, in 1956 [37]. In the following decades, AI experienced several ups and downs [40]. Currently, AI is a broad and thriving field with many applications in practice and several active research topics [41,42,43]. Advancing computing power and an increasing amount of data are among the main reasons for the growing interest in AI in today’s business environment and society [40]. Although AI is often considered to belong to computer science, it is a multidisciplinary field that contains contributions from other disciplines, such as psychology, mathematics, and neuroscience [44,45].

It is important to note, however, that today’s AI systems are not intelligent in the proper sense of the word. In that regard, [46] was the first to propose a distinction between strong and weak AI. Strong AI, or artificial general intelligence (AGI), describes machines or systems that have human-like intelligence or capabilities [47,48]. These strong AI systems might have emotions, feelings, and an understanding of their environment [44]. However, strong AI is not yet realized [49], and some researchers even believe that AI will never be capable of all human abilities [50]. The AI methods and applications of today are examples of weak AI. Weak AI systems are not generally intelligent and are developed for single tasks. They do lack emotions, feelings, general intelligence, or a conscious mind [44,46]. Therefore, weak AI systems are not intelligent, but only behave as though they are [37,51].

AI does not refer to one single tool or application; it is an umbrella term that describes several different technologies. Machine learning is a group of AI methods that is nowadays probably used most often. Nowadays, machine learning is driving many applications in modern life. Examples include web searches, content filtering in social networks, or recommendations on e-commerce websites. Additionally, machine learning is part of many consumer products, such as cameras and smartphones [52]. In general terms, machine learning refers to applications and systems that are able to automatically detect meaningful patterns in data [45]. That means that the performance of machine learning improves with “experience” [53], also referred to as learning or training of machine learning systems. Based on the how the machine learning system learns, two different types can be identified: those with supervised and unsupervised learning techniques [54]. It must be noted that other types of learning exist. Examples are, among others, semi-supervised learning, online learning [55], or reinforcement learning [56,57]. However, supervised and unsupervised learning are the most used learning algorithms [58]. The difference between supervised and unsupervised learning is the presence of labels in the data that are used for training [58]. With supervised learning, the system receives labeled input as the training data [59]. In comparison, in unsupervised learning, a system only receives input but does not obtain information about the desired outcomes [60].

There are a large number of different algorithms that are subsumed under the term machine learning. Among these are tree-based methods, such as the decision tree, random forest, and XGBoost, as well as methods that are inspired by the human brain, such as neural networks [42,61].

The remainder of this article is structured as follows. The second section is divided into three subsections and presents the findings and results of our bibliometric analysis. After that, section three contains a discussion that consists of a future research agenda and implications of our study for research and practice. This is followed by Section 4, in which the bibliometric approach and steps of data collection and analysis are discussed. Finally, the last section contains concluding remarks.

## 2. Results

This section presents the findings of the bibliometric analysis. It consists of three subsections. First, a general overview of the field of AI for drug discovery is given. The second subsection shows the results of our performance analysis. Finally, the last subsection contains a network analysis and a thematic overview.

### 2.1. General Overview

This section gives an overview of the research field of AI applied for drug discovery. Table 1 shows an overview of key metrics of the identified publications. In total, 3884 different documents were identified that dealt with that topic. These documents were published in 1073 different journals or conferences. In total, 217,668 references were cited by the 3884 documents. In total, 6790 different author keywords were used. Apart from the author keywords, which are provided by the original authors of a document themselves, keywords plus are an additional way to analyze a document’s content. Keywords plus are automatically generated and are words or phrases that appear in the titles of an article’s references [62,63]. In summary, 19,326 different keywords plus were identified in our sample.

In total, 12,044 different researchers authored the 3884 articles dealing with AI-based drug discovery. A total of 19,011 authors appear in the publications, and this is equivalent to an average number of 4.89 authors per document. Only 261 documents are single-author papers, and this is equivalent to 6.7% of the articles. This might be an indicator of the topic’s high complexity, which makes it necessary to collaborate with other researchers. This assumption is underpinned by the high number of almost five researchers authoring one document on average. To analyze the cooperation among researchers, the collaboration index (CI) is an often-used variable. It is calculated by dividing the total number of authors of multi-authored documents by the total number of multi-authored articles [64,65]. For our sample, we received a collaboration index of 3.26. This is a high value compared to other bibliometric studies (see Table 2 for a comparison).

**Figure 1:**
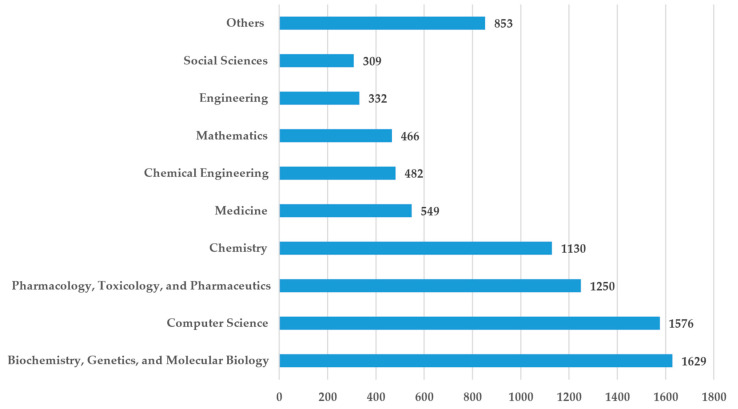
Distribution of the publications among disciplines.

Figure 1 shows the distribution of the identified publications among the different disciplines. The data for Figure 1 were derived from Scopus, where the publications are assigned to disciplines based on the outlets they were published in. An outlet can be related to more than one discipline. Therefore, the total number of documents in Figure 1 is higher than the number of identified documents.

In total, the 3884 identified documents cover 26 different disciplines. The fact that scholars from many different disciplines contributed to it shows the interdisciplinary nature of this research field and AI in general [61]. We can see that most articles have been published within four disciplines. Three of these disciplines are directly related to medicine and topics related to drug development, namely “Biochemistry, Genetics, and Molecular Biology”, “Pharmacology, Toxicology, and Pharmaceuticals”, and “Chemistry”. The fact that “Computer Science” is the discipline with the second-highest number of publications is not surprising, since AI is a traditional topic within computer science. In comparison, “Biochemistry, Genetics, and Molecular Biology” and “Pharmacology, Toxicology, and Pharmaceuticals” are fields that are concerned with discovering and developing new drugs.

**Figure 2:**
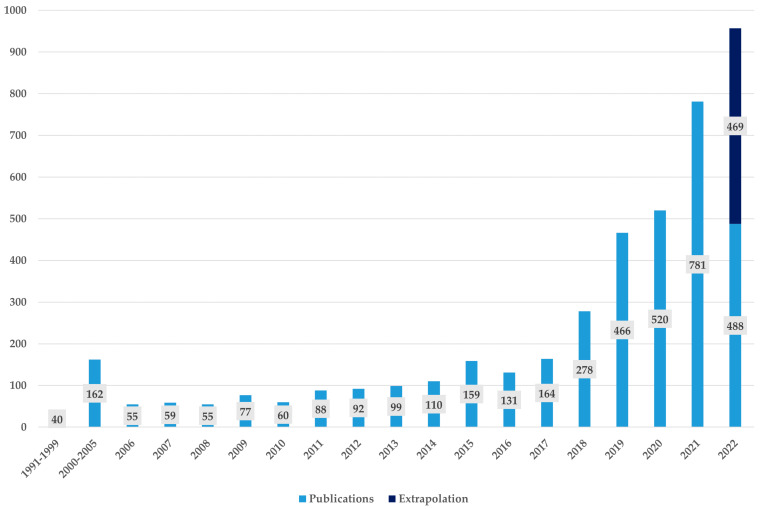
Number of publications per year.

Figure 2 depicts the number of publications for every year. The first identified publication dealing with AI for drug discovery was published in 1991 [70]. In this article, the authors present their preliminary results about the application of machine learning computer-aided molecular design. In this early work, the machine learning that is used is trained with a knowledge base of chemical properties. The goal of the model was to automatically identify relevant fragments in a molecule that are “responsible for activity in a set of inhibitors of thermolysin and, furthermore, to determine a generalized model for an optimal inhibitor” [70].

In the next years, however, we observe only slow growth of research on AI for drug discovery. In total, only 40 articles were published between 1990 and 1999. From 2000 on, there was an increasing interest in the field of AI applied for drug discovery. From 2000 until 2005, four times as many articles were published than in the 10 years before. By the year 2006, the annual number of research is steadily increasing, with the exception of only three years (2008, 2010, and 2016). In the first eight months of 2022, 488 articles were published. We therefore can assume that the trend of an increase in publication numbers will be ongoing in 2022. Since, in previous years of our final sample, statistically more publications appear in the last months of the year, we extrapolated the total number of publications for 2022 to 957 in total.

In summary, Figure 2 shows that the research interest in AI for drug discovery has increased significantly over the last few years. It has to be noted, however, that this development is not unique to AI applications for drug discovery. Instead, AI in general is a topic that has gained a lot of attention in the last years, notwithstanding the application.

### 2.2. Performance Analysis

This subsection presents the results of our performance analysis, while the previous subsection aimed to give a general overview. The 3884 publications that were included in our final sample were published in 1073 different sources. From these sources, 263 were conferences and 810 were journals. Table 3 lists the outlets with the most publications on AI applied for drug discovery. The leading journal with the most articles (*n* = 222) on this subject is the *Journal of Chemical Information and Modeling*. This outlet is followed by the *Briefings in Bioinformatics*, *Drug Discovery Today*, *BMC Bioinformatics*, and the *Journal of Cheminformatics*. Among the 20 sources with the most publications, only one item referred to conference proceedings (*Lecture Notes in Computer Science* on rank 10). The thematic focus of the sources reflects the strong dominance of computer science and biochemistry that was already outlined above (see Figure 1).

AI for drug discovery is a topic of international and global interest. In total, researchers from 100 different countries have contributed to the studies that were identified. In Table 4, the 20 countries with the most publications are listed. A publication is assigned to a country when its corresponding author is affiliated with an institution or company located in this nation. If the corresponding author was not clearly identifiable, an article was excluded from the analysis. In total, 3236 of the identified 3884 articles had a clearly defined corresponding author.

Authors from the United States have authored, by far, the most publications. In total, 850 of the 3236 publications have corresponding authors from institutions or companies in the United States, which is equal to more than 26%. The United States are followed by China, with 577 publications. In summary, authors from China and the United States corresponded to 44% of all publications dealing with AI for drug discovery. China and the United States are followed by India (216 publications) and the United Kingdom (207 publications). With 152 publications, Germany is the first country from the European Union to appear in our list, on the fifth rank.

The United States is also leading in terms of the total citation count. In summary, the articles with a corresponding author from the United States received 69,461 citations, which is equal to an average citation count of 81.72 per document. It is important to mention, however, that one single article, dealing with deep learning in general [52] is responsible for 37,560 of the 69,461 citations of the United States, and this is equal to more than 54%. In terms of the total citation count, the United States is followed by China, the United Kingdom, Germany, Switzerland, and Canada. Additionally, it is interesting to observe that a high number of articles does not necessarily correlate with a high number of citations. When we focus on the average number of citations per document, the United States is followed by Canada, Switzerland, and Singapore.

Next, we take a look at the sponsors that funded most of the articles. Table 5 shows an overview of the top 20 funding sponsors of all articles. Of the 3884 sources we identified, 2003 were supported by funding sponsors. Although the majority of the 2003 articles was funded by only one sponsor, several projects were supported by more than one institution. We can observe that funding sponsors from China, the United States, and the European Union are most often present on the list. Of the top 20 sponsors, 8 were from the United States, four from China, and three from the European Union. The National Natural Science Foundation of China funded the most articles of our sample (361 publications), followed by the National Institutes of Health (327 publications, United States), and the National Science Foundation (153 publications, United States). While most of the funding sponsors belong to one single country, three funding programs were from the European Union, namely the Horizon 2020 Framework Programme, the European Commission, and the Seventh Framework Programme.

The following Table 6 shows the top 20 funding sponsors of the 100 most cited articles of our sample. With 13 funding sponsors, a majority of the 20 top funding sponsors originate from the United States. Furthermore, two funding sponsors from Switzerland are among the top 20, namely the Eidgenössische Technische Hochschule Zürich and the Schweizerischer Nationalfonds zur Förderung der Wissenschaftlichen Forschung. Switzerland is thus the only country in Table 5, apart from the United States, that is represented more than once. The Horizon 2020 Framework Programme is ranked 6th and funded 3 of the 100 most cited articles on AI-supported drug discovery. The National Natural Science Foundation of China, which funded the most articles in total, funded four of the most cited articles.

Finally, our performance analysis consists of an overview of the most productive affiliations. To address this, Table 7 shows an overview of the 20 most productive affiliations. In total, the 12,044 authors that contributed to the research about AI-based drug discovery came from 2970 different affiliations. With 138 authors, the University of California, in the United States, was most often represented in research about AI for drug discovery. The University of California is followed by the Zhejiang University, the Central South University (both located in China), and the University of Cambridge (United Kingdom). From ten affiliations, more than 100 authors contributed to research on AI for drug discovery. The Uppsala University from Sweden and the National University of Singapore are the two only affiliations that are not located in the United States, China, or the United Kingdom.

### 2.3. Science Mapping and Thematic Analysis

Next to the performance analysis that was presented in the last section, we conducted science mapping to obtain a better understanding of the topics and structure of the research field of AI in drug discovery. Together with performance analysis, science mapping is one of the two main categories of bibliometric tools [22]. In contrast to performance analysis, which aims to measure performance, science mapping examines interactions and relationships of research constituents [22,71,72]. Science mapping is a widely adopted set of methods that aims to shed light on a research field’s conceptual, social, and intellectual structure [73,74,75]. By conducting science mapping and studying keywords and their frequency, this section aims to analyze the key topics addressed in research on using AI for drug discovery.

We begin our examination by looking at commonly used keywords that appeared in the title, abstract, or keywords. In Table 8, we show the keywords that appeared most frequently throughout our sample. Many of the most frequently used terms are not surprising since they also occurred in the search string that was used for the literature collection (e.g., drug discovery, machine learning, artificial intelligence, deep learning, or artificial neural network). It is worth mentioning that “machine learning” is the technical term that appeared most frequently in our sample. This is not surprising, since “machine learning” is an umbrella term that often includes frequently used technologies such as decision trees and artificial neural networks [76]. Deep learning is another concept that belongs to machine learning and is based on artificial neural networks [77]. It is therefore hardly surprising that also deep learning is among the top 10 keywords, with 812 appearances of that term in total.

With 1651 and 1391 appearances, the terms “human” and “humans” are also among the top five keywords within our sample. This indicates that a high percentage of the identified research deals with drugs or proteins that are relevant for the human organism. Typical research contributions within this category are machine learning and AI approaches for predicting the interactions between SARS-CoV-2 and human proteins [78], the investigation of human intestinal drug absorption for drug discovery [79], or for the development of G-protein-coupled receptor (GPCR) agonists [80].

**Figure 3:**
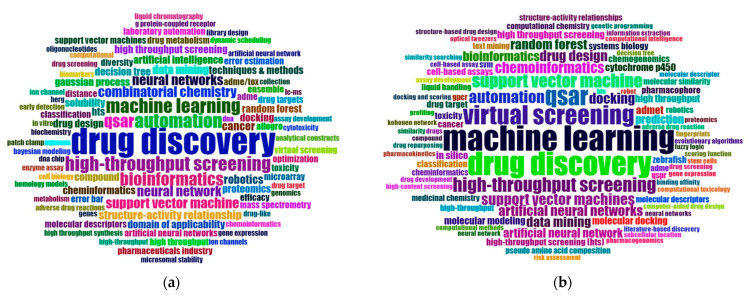
Word clouds of the most frequently appearing author keywords: (**a**) years 1991–2007 (316 articles) and (**b**) years 2008–2013 (471 articles).

**Figure 4:**
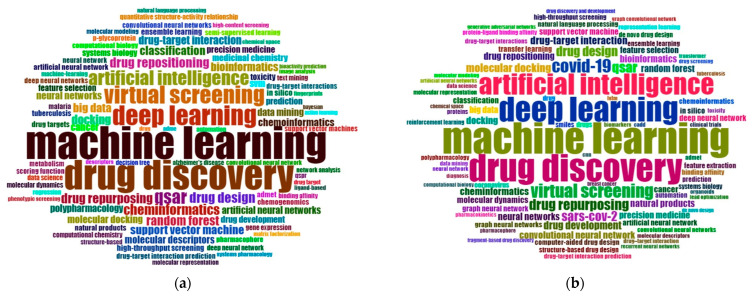
Word clouds of the most frequently appearing author keywords: (**a**) years 2015–2019 (1197 articles) and (**b**) years 2020–2022 (1789 articles).

Figure 3 and Figure 4 show word clouds from different time periods. The bigger a word is, the more often it appeared throughout the keywords of the articles within that period. We divided our sample into four different periods (see Table 9 for an overview). Figure 3a shows the author keywords used most frequently from 1991 to 2007. This is the longest period of time, and it contains 316 articles in total. It is the earliest stage of research on AI for drug discovery. We see that “drug discovery” is by far the most dominant term. When we focus on technology-related terms (e.g., artificial intelligence, machine learning, random forest, etc.), it is interesting to see that there is no clear dominance of one single technology in that early phase.

While machine learning and deep learning seem to dominate the discussions in later periods, many technologies have an equal size in Figure 3a. This shows that researchers were experimenting with different technologies in the early years. Already in the second period that consists of 580 articles published between 2008 and 2014, machine learning has become the technology most often used, followed by support vector machines.

Figure 4 shows the word clouds from the most recent periods. Figure 4a contains 1197 articles published between 2015 and 2019. In the third period, machine learning is obviously the most often used author keyword and the most popular group of technologies for drug discovery within our sample. While support vector machines were still frequently applied in the second period (Figure 3b), they lost a lot of attention from 2014 on. Furthermore, the third period is characterized by the rise of deep learning which is now appearing among the keywords for the first time. In 2015, the first three articles were published that suggested or investigated deep learning’s potential for drug discovery [52,81,82]. From 2015 on, research containing deep learning grew continuously. In 2016, 10 more articles on deep learning for drug discovery were published, followed by 18 publications in 2017, 58 publications in 2018, and 138 publications in 2019.

Given that tremendous growth, it is not surprising that “deep learning” is among the top keywords in the latest period (Figure 4b). In total, 644 publications containing “deep learning” as an author keyword were published between 2020 and 2022. Together with “machine learning” and the umbrella term “artificial intelligence”, “deep learning” is thus the most frequently applied technology for drug discovery in the last few years. Additionally, COVID-19 and SARS-CoV-2 are now frequently used author keywords. As recent studies show, machine learning or AI can be used for several for the discovery and development of drugs or antibodies against COVID-19 [83]. For example, [84] proposes a deep learning model for screening effective inhibitors against SARS-CoV-2, while [85] presents D3AI-CoV, which is a platform that consists of three deep learning models that aim to support the discovery of drugs against COVID-19. These examples show that AI-based technologies can help to quickly understand new diseases and to find countermeasures against them.

**Figure 5:**
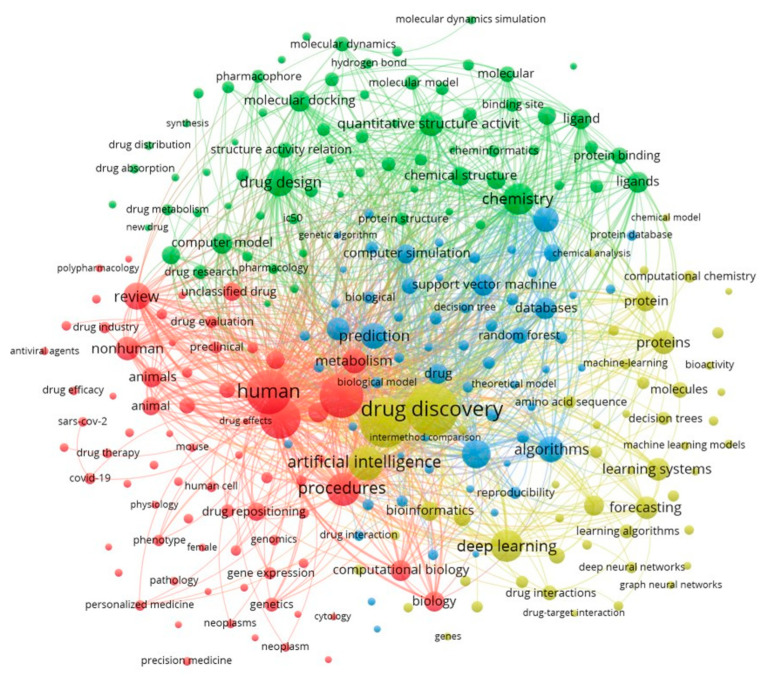
Keyword co-occurrence of the most-used author and indexed keywords.

Finally, Figure 5 shows a graphical representation of the keyword co-occurrences. The underlying assumption of a co-word analysis is “that words that frequently appear together have a thematic relationship with one another” [22] (p. 289). In Figure 5, only keywords appear that were used at least 50 times in our sample. Similar to word clouds, terms that occur more frequently are represented with a bigger font size and circle. Terms that appear together are linked with lines. Following the same logic as the font and circle size, a line between two terms is thicker the more often these two terms appeared together in one publication. Additionally, words that are in the center of the network are linked to other words in different clusters. Keywords that are less linked and do not have many relations to other clusters are depicted at the edge of Figure 5.

When we look at Figure 5, we can see four different thematic clusters that are represented in different colors. First, the red cluster is the medical branch of the research that deals with AI for drug discovery. Keywords such as “human”, “nonhuman”, or “animal” indicate that the focus is on understanding drugs in relation to organisms. The abovementioned works that deal with the use of AI for the development of drugs to fight diseases such as COVID-19 [83,84,85] are typical examples. Keywords such as “personalized medicine”, “precision medicine”, or “human cell” indicate that also the development of tailored drugs is part of this cluster, for example, to better fight cancer [86,87].

The green cluster consists of keywords such as “chemistry”, “drug design”, or “chemical structure”. While the green cluster is also about drug design, a keyword such as “chemical structure”, “molecular model”, or “molecular dynamics” indicates that the green cluster is more concerned with topics that belong to chemistry. Examples that belong to this cluster are the application of AI to understand molecular docking to discover and design marine drugs [88] or the prediction of molecular properties [89]. In most cases, the ultimate goal of these articles within the green cluster is still about developing drugs that can be used for treatment. However, the method and approach that underlies the green cluster’s articles are often different.

While the red and green clusters are driven by medicine and natural sciences, such as biology and chemistry, the yellow cluster is more technology oriented. In this cluster, many technologies, such as machine learning, learning systems, and deep neural networks, are among the keywords. Articles within this cluster often do investigate the potential of AI for drug discovery from a more technical point of view. Closely related to the yellow cluster is also the blue group of terms. The blue cluster is located most centrally, and its terms have several relationships to all the other thematic areas. It is therefore hard to identify the thematic core of the blue cluster and to distinguish it from the from the other ones. In essence, publications within the blue cluster investigate the topic of AI for drug discovery more theoretically. Typical examples that fall within the blue cluster are review articles, for example, on computational model development of drug–target interaction prediction [90], molecular docking [91], or how AI applications can be combined with other approaches for drug discovery [92]. Overall, the keyword “co-occurrence network” in Figure 5 underpins the multidisciplinary nature of AI in the context of drug discovery.

## 3. Discussion

### 3.1. Future Research Agenda

We presented the state of research in the previous section, and the following section derives possible research questions that have not yet been addressed to further advance AI-based research in drug discovery.

First, considering the word clouds in Figure 3 and Figure 4, it becomes evident that the focus of studies that use AI for drug discovery has changed significantly. While studies in the 1990s generally associated AI with automating drug discovery, the focus gradually concretized toward machine learning. It is noteworthy that most of the applied algorithms in the studies belong to the field of supervised learning. Algorithms such as support vector machines [93,94], random forests [95,96], and neural networks [97,98] are applied to already known drug discovery problems in order to identify the most important influencing variables based on the given input data. However, the limitations of supervised learning are that it can only be applied to classification problems that are already well known. For drug discovery, this means that supervised learning models require a large amount of labeled data from the past to identify patterns for an already known drug discovery problem. These patterns can be extrapolated to similar problems but are not transferable to new, completely unknown drug discovery problems. For this purpose, the use of unsupervised learning algorithms, such as clustering methods, would be necessary. Examples of clustering methods are principal component analysis [99,100], k-nearest neighbors [101,102], or autoencoder [61,103]. Therefore, we would like to encourage future researchers to increasingly use and evaluate unsupervised learning algorithms to identify patterns in unlabeled data to address unknown drug discovery problems.

Another important topic that future research needs to investigate is the explainability of AI algorithms. Most of today’s best-performing machine learning models are not capable of conveying information about how they came up with their results and predictions. To human users, these machine learning algorithms, therefore, are black boxes [104]. Although some AI researchers argue that explainable AI (XAI) might not be necessary or too difficult to achieve, there are use cases where a certain degree of explainability might be necessary. This is especially important for users to trust and understand AI systems in critical applications such as law, defense, and also medicine [105,106,107]. For drug discovery, XAI is of high importance as well. The authors of [108] state that the explainability is important to validate and understand the results and may have a great impact on the drug discovery pipeline. Furthermore, [109] argues that medical decision-making without any reasoning or justification may contravene the moral responsibilities of clinicians. Although the first research exists on how XAI can be realized in drug discovery [108,109,110], the explainability of many complex models is not yet realized to a satisfying extent. Sometimes, given the challenges research is faced with to realize XAI, it is sometimes argued that more simple models with less predictive power but a higher degree of explainability should be used [109,111]. Therefore, one can argue that there is a trade-off between the predictive power and explainability of AI models. It is, therefore, necessary to investigate if predictive power and accuracy or explainability are more important for AI in drug discovery.

Table 10 below provides an overview of our open research agenda to assist future researchers to further contribute to AI-based drug discovery research. We consider both qualitative and quantitative research methods to adequately address the research gaps. In order to identify the current state of practice, surveys and interviews are suitable methods. Analogously, the current state of research can be demonstrated through a systematic review of the literature. In contrast, for identifying a more effective or efficient solution to existing practical problems, design science research is more appropriate, as different approaches are applied and evaluated until a sufficient practical solution is achieved [112,113]. Another method that can be applied for the evaluation of new approaches involves experiments that analyze existing and potential cause-effect relationships through an isolated perspective [114].

One method that might be particularly suitable to advance research is design science research (DSR). DSR is a method that is commonly used in engineering and information systems research. In general, DSR is concerned with the design of artifacts for an identified problem [115,116]. Valuable results of DSR can be of different kinds and include both newly designed sociotechnical artifacts and design knowledge that explains why certain artifacts are valuable for a given context or application [112,113]. The design of an artifact can be iterative and involve different steps, such as validity tests, evaluation, or experimentation [112]. Right now, DSR is a methodology that has not been applied within research on AI applied for the discovery and development of drugs. This is surprising since AI systems can be understood as technical artifacts. Given that, DSR might be a fitting research paradigm that could guide researchers to conduct and present their research results on AI-supported drug discovery. Although DSR might be uncommon to most researchers outside of information systems and engineering, we, therefore, see a promising chance in applying this method to that research field. This could also be realized by cooperating with information systems or engineering scholars that are familiar with DSR.

### 3.2. Implications

By conducting a bibliometric analysis, we aimed to provide a holistic picture of research dealing with the application of AI for drug discovery. To do so, we conducted a performance analysis, showed the current development of key topics, and presented promising future research avenues.

Our results have several implications for both research and practice. First, our results can help interested researchers and scholars to gain an initial understanding and overview of the research dealing with AI methods in the context of drug discovery. This involves the current development of this area, as well as the most investigated topics and technologies. Additionally, we pointed out several areas that might be addressed by future researchers to help better understand the benefits, challenges, and implications of AI for drug development. As the results and the future research area show, AI for drug discovery is a research field characterized by a high degree of interdisciplinarity. There are still several gaps that require interdisciplinary teams and contributions from many different disciplines. Future research projects must therefore not be limited to scholars that belong to computer science, medicine, or pharmacology. Instead, contributions can be made by researchers from several disciplines.

Apart from researchers, practitioners in medicine or pharmacy can also use our findings to get familiar with AI and its potentials. Not only the clinical practice can use our findings, but also companies. AI not only can be used for existing processes and tasks within companies, but it can also form the foundation for new business models or start-ups [117]. Within the field of AI for drug discovery, too, start-ups emerged that aim to develop new solutions. Entrepreneurs and companies that are interested in entering the market of AI for drug discovery can use this article’s findings to gain an overview of relevant topics and technologies used.

Our study might be subject to certain limitations. First, we used only Scopus as the scientific database for our data collection. Although Scopus is among the most often used databases and covers a large number of relevant outlets, it is likely that not all publications that deal with AI for drug discovery are included in our sample. However, given the large number of databases and outlets Scopus covers, we believe that this does not affect the main findings of our study significantly. Nevertheless, it might be possible that the usage of other databases might lead to slightly different results. Furthermore, AI is under rapid development, and the number of articles investigating its use for drug discovery is continually rising. Therefore, given the unpredictability of technological developments in the future [118], the results of this article can only represent the current state of the research. It is possible that new trends and technologies will appear that are not covered by this analysis. Finally, we did not use all methods that belong to the methodological toolbox of bibliometric studies. There are other techniques, such as citation analysis [119], co-citation analysis [120], or bibliographic coupling [121,122], that are not part of this study. The application of other methods might therefore lead to additional insights.

## 4. Materials and Methods

The following section describes the research method applied. The bibliometric approach we used can be divided into two parts. First, the bibliometric data were collected from the database Scopus. This step is explained in the first subsection. After that, the exported data were analyzed, as explained in the second subsection.

### 4.1. Data Collection

The first step of a bibliometric study is the collection of metadata that serve as the foundation of the analysis [123]. Many bibliometric databases exist that differ in terms of their functionalities and characteristics (for an overview, see [124]). Following the recommendation of [22], we decided to rely on only one single database. The reason for this decision is that every database has its own format of bibliometric data efforts to combine different formats from different databases, and this can easily lead to errors [22]. Although many bibliometric databases do exist, Scopus and the Web of Science (WoS) are among the most relevant ones [69,73]. Compared to the WoS, however, Scopus covers more scientific journals [21]. Like the authors of other recent studies (see, e.g., [23,24,125,126]), we, therefore, chose Scopus as the database for our data collection.

Regarding the terms for our search string, we used [6] as a reference. Apart from general terms such as “artificial intelligence” or “machine learn*”, we also searched for more specific technologies, such as “fuzzy expert system” or “evolutionary computation”. The search string’s second part consisted of the application we searched for, namely drug detection. Apart from “detection”, we used “discovery” as a synonym. However, it should be noted that studies dealing with the identification of illicit drugs are not part of the final sample, as there is no intersection with drug discovery research. This resulted in the following search string that was applied in Scopus to search the title, abstract, and keywords:

((“Artificial intelligence” OR “Machine intelligence” OR “artificial neural network*” OR “Machine learn*” OR “Deep learn*” OR “robotic” OR “thinking computer system” OR “fuzzy expert system*” OR “evolutionary computation” OR “hybrid intelligent system*”) AND (“drug detection” OR “drug discovery”))

**Figure 6:**
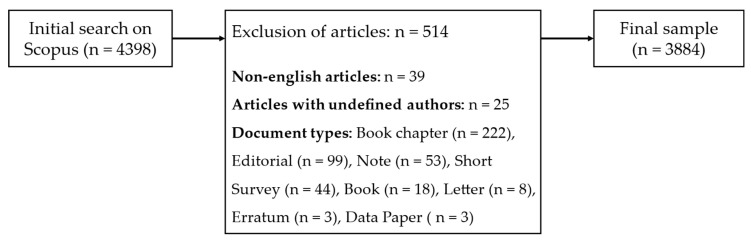
Overview of the data collection and the exclusion of articles.

We received 4398 documents as the first result. After the initial search, documents were excluded based on different criteria. First, only English articles were further considered. This led to the elimination of 39 documents. After that, 25 articles with undefined authors were removed from the sample. Finally, we excluded documents based on their type. In this step, only documents that were reviews, journal articles, or conference submissions remained. These steps led to a final sample of 3884 documents that were further considered. The overall process is depicted in Figure 6.

Finally, the remaining sample of 3884 articles was imported in the CSV format for further analysis. This process of data analysis is explained and outlined in the following subsection.

### 4.2. Data Analysis

A broad variety of different tools exist that can help us analyze bibliometric data. For our bibliometric analysis, we followed [69] and used two different tools in combination: VOSviewer and Bibliometrix/Biblioshiny.

VOSviewer is a tool that was developed by the Centre for Science and Technology Studies at Leiden University in the Netherlands [124,127]. VOSviewer is a widely known application that has been applied in numerous bibliometric studies. It supports the construction of bibliometric networks consisting of publications, journals, or researchers. Furthermore, it allows for co-citation, bibliographic coupling, and co-authorship analysis [124,127]. Given these advantages, we decided that VOSviewer is a useful tool for our study. Next to VOSviewer, we used the open-source R package Bibliometrix, which was developed by [128]. Additionally, we complemented Bibliometrix with Biblioshiny. Biblioshiny is a tool for the creation of bibliometric visualizations and analyses [124]. We used Bibliometrix and Biblioshiny since these enable many different types and forms of analysis [124]. Although Biblioshiny offers many different functionalities for the statistical analysis of bibliometric data, we decided to complement it with VOSviewer since, as outlined above, this tool is highly suitable for visualization purposes [69].

## 5. Conclusions

AI is a group of technologies that are nowadays used and investigated for several use cases. In the context of drug discovery, the usage of AI technologies is a topic of rising interest. Due to its growth in the last years, AI for drug discovery is a research field that consists of many different topics and articles. These contributions stem from researchers from several countries and institutions. Due to the high number of contributions, the research field on AI for drug discovery became increasingly complex and difficult to oversee. With this article, we aimed to address this increasing complexity by giving an overview of research on AI for drug discovery as a whole. To do so, our first research goal was to investigate the present status of research on using AI for drug discovery and what topics have been investigated in previous research. To do so, we conducted a bibliometric analysis that consisted of 3884 articles published between 1991 and 2022. We applied a performance analysis to identify the most productive institutions, countries, and funding sponsors. Additionally, we used science mapping and thematic analysis to identify the core topics and thematic areas.

Furthermore, it was our second goal to identify promising future research avenues that can help to advance the field of AI for drug discovery. Based on the results of the bibliometric study and content analysis, we outlined different directions, research questions, and exemplary methods that can be applied by future scholars. Both the findings and the future research opportunities indicate the multidisciplinary nature of AI and its applications for understanding and discovering drugs. We hope that, with our findings and overview, we have succeeded in providing a good foundation for interested researchers and scholars from all disciplines to investigate this interesting and exciting topic.

## Figures and Tables

**Table 1:** General overview and key metrics of the identified publications.

Metric	Value
**Main information**	
Timespan of publications	1991–2022
Sources (conferences and journals)	1073
Documents	3884
Average citations per document	36.08
Average citations per year per document	5.70
Total number of references	217,668
Number of author’s keywords	6790
Number of keywords plus	19,326
**Document Types**	
Journal article	2452
Conference article	503
Review	928
**Authors**	
Number of different authors	12,044
Total number of author appearances	19,011
Average number of authors per document	4.89
Documents per author	0.322
Single-authored documents	261
Multi-authored documents	3623
Authors of multi-authored documents	11,803
Collaboration index	3.26

**Table 2:** Comparison of different bibliometric studies.

Study	[66]	[67]	[68]	[69]	This Study
Topic	Data quality	Blockchain in accounting	Waqf research	Data governance	AI for drug discovery
Documents	159	93	527	780	3884
Documents per author	0.305	0.443	0.599	0.367	0.322
Collaboration index	3.60	2.83	2.53	3.26	3.26
Single-authored documents	-	29%	50%	22.18%	6.7%

**Table 3:** Overview of the sources with the most publications.

Rank	Source	Publications
01	*Journal of Chemical Information and Modeling*	222
02	*Briefings in Bioinformatics*	95
03	*Drug Discovery Today*	85
04	*BMC Bioinformatics*	71
05	*Journal of Cheminformatics*	70
06	*Molecules*	70
07	*International Journal of Molecular Sciences*	69
08	*Expert Opinion on Drug Discovery*	64
09	*Bioinformatics*	57
10	*Lecture Notes in Computer Science*	56
11	*Journal of Computer Aided Molecular Design*	55
12	*Molecular Informatics*	53
13	*Scientific Reports*	52
14	*Molecular Pharmaceutics*	46
15	*Current Topics in Medicinal Chemistry*	44
16	*PLOS One*	44
17	*Journal of Medicinal Chemistry*	39
18	*IEEE ACM Transactions on Computational Biology and Bioinformatics*	36
19	*Molecular Diversity*	36
20	*Frontiers in Pharmacology*	33

**Table 4:** Overview of the countries with the most publications (*n* = 3236).

Rank	Country	Publications	Percentage	Citations	Avg. Cit. per Document
01	USA	850	26.27%	69,461	81.72
02	China	577	17.83%	11,290	19.57
03	India	216	6.67%	3496	16.19
04	United Kingdom	207	6.40%	6517	31.48
05	Germany	152	4.70%	5601	36.85
06	Japan	106	3.28%	1588	14.98
07	Switzerland	90	2.78%	4965	55.17
08	South Korea	87	2.69%	1380	15.86
09	Canada	82	2.53%	4817	58.74
10	Italy	69	2.13%	2199	31.87
11	Spain	62	1.92%	2400	38.71
12	France	60	1.85%	967	16.12
13	Iran	58	1.79%	729	12.57
14	Sweden	53	1.64%	1804	34.04
15	Brazil	49	1.51%	1165	23.78
16	Australia	41	1.27%	719	17.54
17	Portugal	40	1.24%	920	23.00
18	Singapore	30	0.93%	1314	43.80
19	Turkey	30	0.93%	698	23.27
20	Netherlands	24	0.74%	925	38.54

**Table 5:** Overview of the funding sponsors.

Rank	Funding Sponsor	Country/Region	No.
01	National Natural Science Foundation of China	China	361
02	National Institutes of Health	United States	327
03	National Science Foundation	United States	153
04	National Institute of General Medical Sciences	United States	119
05	National Key Research and Development Program of Chinas	China	83
06	U.S. Department of Health and Human Services	United States	79
07	National Cancer Institute	United States	72
08	National Research Foundation of Koreas	South Korea	68
09	Horizon 2020 Framework Programme	European Union	62
10	European Commission	European Union	59
11	Japan Society for the Promotion of Science	Japan	54
12	National Center for Advancing Translational Sciences	United States	54
13	National Institute of Allergy and Infectious Diseases	United States	46
14	Fundamental Research Funds for the Central Universities	China	41
15	Engineering and Physical Sciences Research Council	United Kingdom	40
16	Ministry of Education, Culture, Sports, Science and Technology	Japan	37
17	U.S. National Library of Medicine	United States	35
18	Seventh Framework Programme	European Union	35
18	Schweizerischer Nationalfonds zur Förderung der Wissenschaftlichen Forschung	Switzerland	34
19	Ministry of Science and Technology of the People’s Republic of China	China	34
20	Deutsche Forschungsgemeinschaft	Germany	33

**Table 6:** Overview of the funding sponsors of the 100 most cited articles.

Rank	Funding Sponsor	Country/Region	No.
01	National Institutes of Health	United States	12
02	National Institute of General Medical Sciences	United States	7
03	National Science Foundation	United States	6
04	National Natural Science Foundation of China	China	4
05	National Heart, Lung, and Blood Institute	United States	4
06	Horizon 2020 Framework Programme	European Union	3
07	Eidgenössische Technische Hochschule Zürich	Switzerland	3
08	Biotechnology and Biological Sciences Research Council	United Kingdom	2
09	Canadian Institutes of Health Research	Canada	2
10	Deutsche Forschungsgemeinschaft	Germany	2
11	Japan Society for the Promotion of Science	Japan	2
12	National Cancer Institute	United States	2
13	National Center for Advancing Translational Sciences	United States	2
14	National Institute of Allergy and Infectious Diseases	United States	2
15	National Institute of Biomedical Imaging and Bioengineering	United States	2
16	NVIDIA	United States	2
17	Pharmaceutical Research and Manufacturers of America Foundation	United States	2
18	Schweizerischer Nationalfonds zur Förderung derWissenschaftlichen Forschung	Switzerland	2
19	U.S. Food and Drug Administration	United States	2
20	U.S. National Library of Medicine	United States	2

**Table 7:** Overview of the most productive affiliations.

Rank	Affiliation	Country/Region	No.
01	University of California	United States	138
02	Zhejiang University	China	129
03	Central South University	China	128
04	University of Cambridge	United Kingdom	128
05	Sun Yat-Sen University	China	122
06	China Pharmaceutical University	China	121
07	Novartis Institutes for Biomedical Research	United States	120
08	Sichuan University	China	118
09	East China University of Science and Technology	China	116
10	University of Pittsburgh	United States	115
11	National University of Singapore	Singapore	89
12	Peking University	China	74
13	National Institutes of Health	United States	72
14	Stanford University	United States	72
15	Tsinghua University	China	72
16	Shanghai Jiao Tong University	China	71
17	University of British Columbia	United States	70
18	Uppsala University	Sweden	66
19	Vanderbilt University	United States	63
20	Shanghai Institute of Materia Medica	China	62

**Table 8:** Overview of the most frequently used keywords.

Rank	Keyword	No.
01	Drug discovery	2127
02	Machine learning	1877
03	Human	1651
04	Drug development	1525
05	Humans	1391
06	Article	1369
07	Artificial intelligence	933
08	Procedures	912
09	Deep learning	812
10	Chemistry	782
11	Priority journal	729
12	Drug design	689
13	Review	689
14	Algorithm	669
15	Nonhuman	558
16	Algorithms	557
17	Prediction	549
18	Metabolism	525
19	Protein	494
20	Quantitative structure activity relation	490
21	Artificial neural network	440
22	Forecasting	432
23	High-throughput screening	426
24	Learning systems	419
25	Animals	417
26	Support vector machine	403
27	Controlled study	387
28	Computational biology	377
29	Ligands	375
30	Drug screening	371

**Table 9:** Overview of the time periods.

Years	Number of Publications	Duration of the Period
1991–2007	316	17 years
2008–2014	580	7 years
2015–2019	1197	5 years
2020–2022	1789	3 years

**Table 10:** Open research agenda.

Focus	Possible Research Questions	Exemplary Research Methods
AI algorithms	Which AI algorithms have the best accuracy for certain tasks in drug detection?	Systematic literature review, design science research
	How promising is the application of unsupervised learning algorithms for drug discovery?	design science research
	Should the explainability or accuracy of AI algorithms be prioritized in drug discovery?	Interviews
	Which prediction accuracy is required to classify the algorithm as reliable for drug discovery?	Experiments, interviews
	How can the explainability of specific algorithms be increased?	Experiments, design science research
Acceptance by pharmacists and customers	What is the current level of acceptance of pharmacists to use AI for drug detection?	Surveys
	Which tasks will be performed by pharmacists and which by AI in the future?	Conceptual, experiments
	Do patients trust drugs developed by AI?	Surveys, interviews
	How can pharmacists be trained for using AI effectively?	Conceptual, experiments
Data management and security	How can AI algorithms for drug discovery be protected against malicious manipulation?	Conceptual, experiments, design science research
	Is there a danger of adversarial attacks and how can these be avoided?	Experiments, design science research
	How should the data be stored to ensure efficient training of machine learning algorithms?	Survey, experiments
	How can the data that is involved be protected against data theft?	Survey, experiments
Law and regulation	How can data privacy regulations, such as HIPAA and GDPR, be ensured?	Conceptual
	Does the drug approval process need to be adjusted in case of an AI-based solution?	Conceptual
	Who is liable in case of a patient’s claims for damages?	Conceptual
	Is AI-assisted drug discovery compliant with the governance principles of the pharmaceutical industry?	Conceptual, interviews
	How could quality assurance processes be ensured for a machine learning algorithm?	Conceptual

## Data Availability

The data presented in this study are available in the article.
